# Computational identification of migrating T cells in spatial transcriptomics data

**DOI:** 10.1172/jci.insight.192718

**Published:** 2026-05-08

**Authors:** Lin Zhong, Bo Li, Zhikai Chi, Siyuan Zhang, Qiwei Li, Guanghua Xiao

**Affiliations:** 1 Quantitative Biomedical Research Center, University of Texas Southwestern Medical Center, Dallas, Texas, USA.; 2 Department of Statistics and Data Science, Southern Methodist University, Dallas, Texas, USA.; 3 Department of Pathology and Laboratory Medicine, Perelman School of Medicine, University of Pennsylvania, Philadelphia, Pennsylvania, USA.; 4 Department of Pathology and Laboratory Medicine, Children’s Hospital of Philadelphia, Philadelphia, Pennsylvania, USA.; 5 Department of Pathology, University of Texas Southwestern Medical Center, Dallas, Texas, USA.; 6 Department of Mathematical Sciences, The University of Texas at Dallas, Richardson, Texas, USA.

**Keywords:** Immunology, Oncology, Cancer, Cell migration/adhesion, T cells

## Abstract

T cells are the central players in antitumor immunity, and effective tumor killing depends on their ability to infiltrate into the tumor microenvironment (TME) while maintaining normal cytotoxicity. However, late-stage tumors develop immunosuppressive mechanisms that impede T cell movement and induce exhaustion. Investigating T cell migration in human tumors in vivo could provide insights into tumor immune escape, although it remains a challenging task. In this study, we developed ReMiTT, a computational method that leverages spatial transcriptomics data to track T cell migration patterns within tumor tissue. Applying ReMiTT to multiple tumor samples, we identified potential migration trails. On these trails, chemokines that promote T cell trafficking displayed an increasing trend. Additionally, we identified key genes and pathways enriched on these migration trails, including those involved in cytoskeleton rearrangement, leukocyte chemotaxis, cell adhesion, leukocyte migration, and extracellular matrix remodeling. Furthermore, we characterized the phenotypes of T cells along these trails, showing that the migrating T cells are highly proliferative. Our findings introduce an approach for studying T cell migration and interactions within the TME, offering valuable insights into tumor-immune dynamics.

## Abstract 2

We developed a computational tool to locate moving T cells in solid tumors and identified key genes involved in this process.

## Introduction

T cell cytotoxicity is essential for the antitumor adaptive immune response (1, 2). After priming by tumor antigens in the draining lymph spots (3), effector T cells travel through the lymphatic vessels to enter the tumor microenvironment (TME) (4). This process is mediated by the T cells expressing necessary cell adhesion molecules and chemokine receptors for migration and infiltration in the tumor (5, 6). Efficient tumor killing hinges on (a) the ability of T cells to infiltrate into the tumor core regions (7) and (b) T cells maintaining a high level of functionality and cytotoxicity. In late-stage tumors, however, either or both processes are disabled, creating a hostile environment that prevents T cell infiltration (8) or rapidly induces T cell exhaustion during their migration inside the tumor region (9).

Clinical efforts have been prioritized to lift T cell exhaustion by immune checkpoint blockade (10, 11) or to break down the tumor barrier to turn immune “cold” tumors “hot” (12). In addition, reduced motility has been observed among exhausted T cells (13). Therefore, investigation on how T cells migrate inside the tumor region and how they become dysfunctional along the way may provide novel therapeutic insights. To date, in vitro experiments have been performed to track T cell movement on 2D or 3D culture conditions, which improved the quantitative understanding of T cell migration (14–17). Noninvasive in vivo tracing of cell movement requires the use of radioactive or fluorescent dyes, which can be monitored with intravital imaging. This has been done using transgenic mouse models with high single-cell resolution (18, 19). However, this approach is usually technically demanding and cannot reach the resolution and scale needed to study T cell migration in the TME. Consequently, there is still a lack of investigation of T cell migration in the TME.

The recent development of the cutting-edge spatial transcriptomics (ST) technologies, such as 10xVisium, Visium HD, MERFISH, Slide-Seq, NanoString CosMx, etc., (20–23), provided an opportunity to address this problem. ST data map the location of gene expression directly within a tissue’s spatial architecture and allow researchers to study gene activity within the context of tissue structure. In this work, we developed what we believe to be a novel computational method, reconstruction of migration trails for T cells (ReMiTT), to identify T cell migration trails using ST data. Previous in vitro studies have demonstrated that when navigating through a 3D collagen matrix, T cells prefer to migrate through less dense or channel-like regions, often following each other along the same trajectory (15). We hypothesize that specific areas with remodeled tumor extracellular matrix (ECM) serve as physical highways facilitating T cell migration. Considering that T cells inevitably become exhausted in solid tumors, an increasing trend of exhaustion levels may be observed along some of these migration trails, where streams of T cells follow each other to enter and traverse these regions. ReMiTT is designed to locate these trafficking T cells by searching for closely located T cell loci or spots that exhibit an increasing trend of exhaustion. This method might provide a snapshot of a stream of migrating T cells on the “physical highway.” Computational methods, such as SpaGCN, BayesSpace, and Giotto (24–26), identify localized spatial domains or coexpression niches by detecting regions with coherent gene expression profiles. However, these approaches are not designed to capture 2D spatial trajectories characterized by directional or progressive transitions in cell states. In contrast, ReMiTT is specifically designed to detect spatially continuous migration trails along which T cells exhibit directional changes in exhaustion status, a feature not captured by domain-based clustering approaches. We implemented this method to study human tumor samples and identified multiple T cell migration trails on a tissue slide. The biological relevance of the trails was validated using T cell chemotaxis markers and T cell receptor genes. Systematic investigations of the genes overexpressed on the migration trails showed enrichment of gene pathways related to T cell migration, chemokine production, cytoskeleton rearrangement, and ECM remodeling on these trails. Furthermore, we identified evidence that there are elevated levels of immunosuppressive cell types, such as Tregs, on migration trails and that density of tumor-associated macrophages (TAMs) increases along the trails. Our study might provide an approach to unbiasedly explore T cell migration patterns and related genes in human cancer samples.

## Results

### Characterization of T cell migration trails.

**Figure 1:**
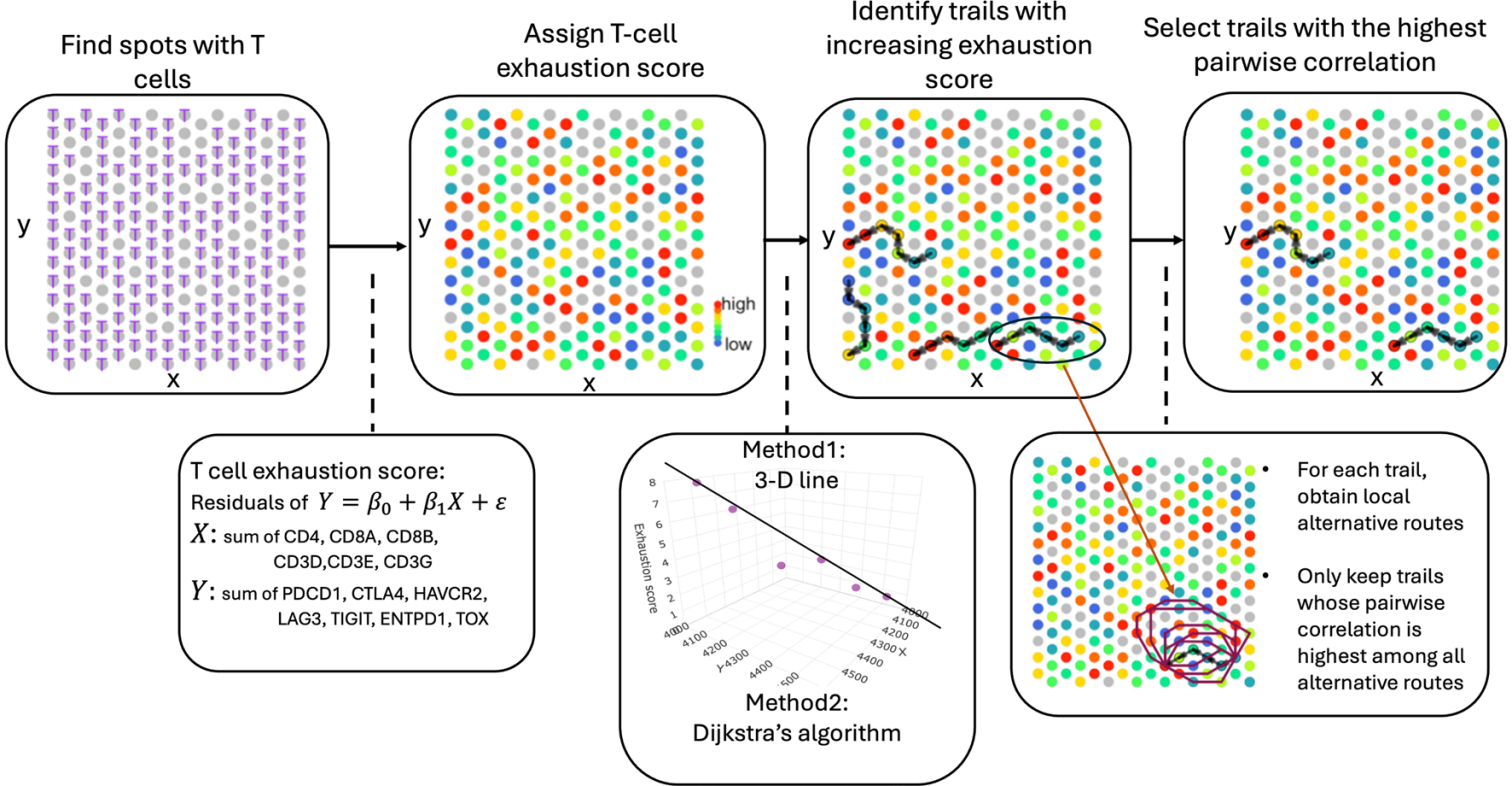
Schematic illustration of the T cell migration trail identification method. We first identified spots with expression of T cell surface markers (CD4, CD8, and CD3) to select those with T cell infiltration. A T cell exhaustion score was then assigned for each T cell spot using putative exhaustion markers. Candidate migration trails were identified by searching for increasing exhaustion scores among adjacent spots. To filter for natural migration trails, we only kept the ones whose mean pairwise correlation among genes associated with T cell stage was higher compared with all its alternative routes.

We implemented ReMiTT to analyze tumor samples profiled using the 10x Visium ST platform from 10x Genomics. This approach is based on a simple observation that migrating T cells in the TME will gradually lose their ability to kill the cancer cells and enter an exhaustion state (27). Therefore, we hypothesized that the continued movement of T cells can be captured by “stitching” neighboring spots on a 2D slide with matched T cell phenotypes; this led to the development of ReMiTT. In brief, we first identified spots that were most likely to contain newly entered T cells from the draining lymph spots as the potential starting points of migration trails of T cells. ReMiTT then searched for 1D trails on a 2D slide, such that T cells on these trails displayed an increasing trend of a predefined score of T cell exhaustion, using either a minimum spanning path searching method or a 3D line-based approach (Figure 1).

**Figure 2:**
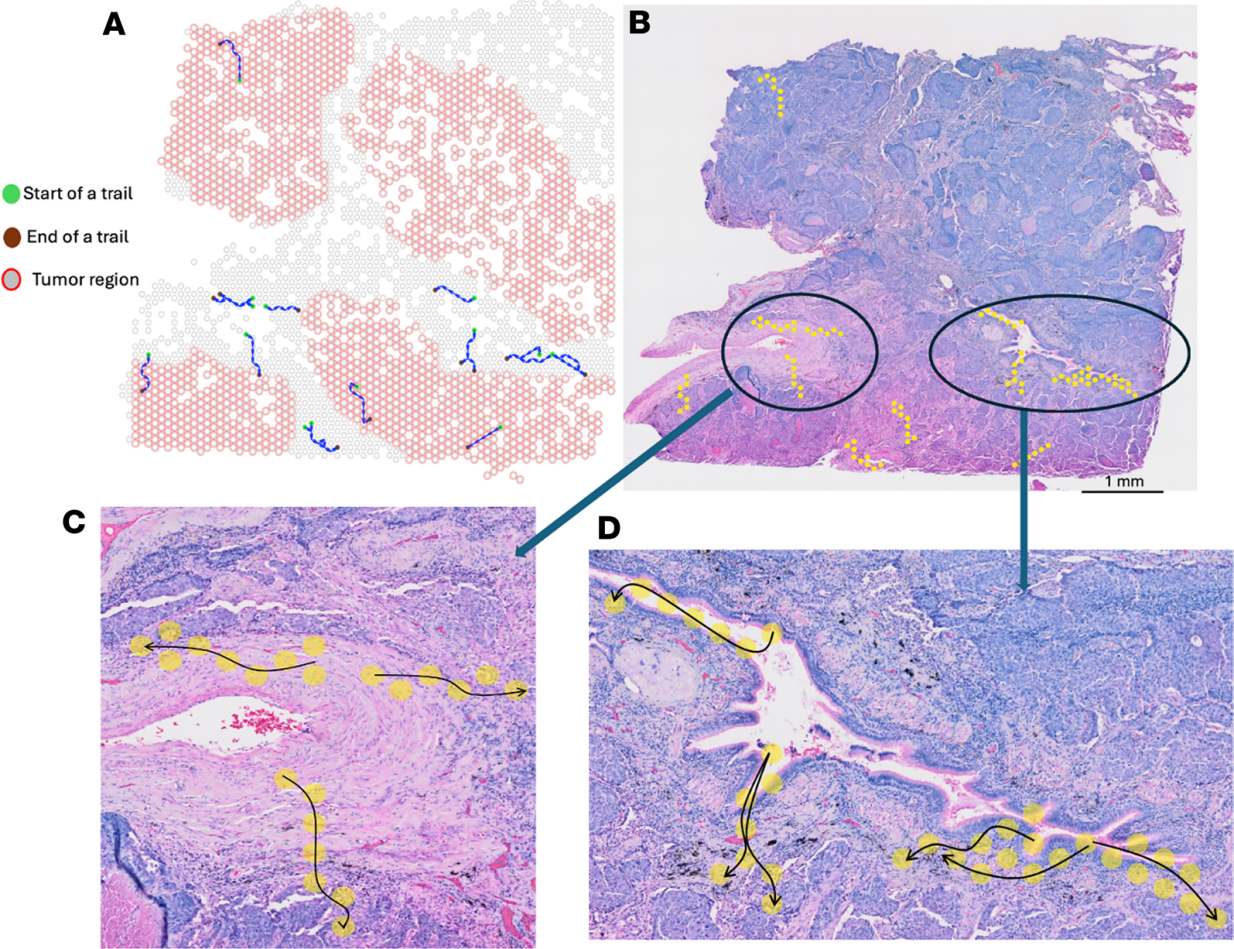
Migration trails identified in a human lung cancer sample and their projections on the pathology slide. (**A**) Algorithm-identified migration trails labeled as splines on the spatial coordinates, with the starting location marked as green. Gray spots represent spots with T cell infiltration, and red circles indicate spots with high levels of expression of lung cancer markers. (**B**) The same trails overlaid on the pathology (H&E) slide of the tumor. (**C**) Examples of trails whose origin is close to vessels. (**D**) Examples of trails that travel in less dense TME.

We applied ReMiTT to a human lung tumor sample, a human ovarian cancer sample, and a human melanoma sample generated with the 10x Visium ST platform. We identified 19 T cell migration trails in the ST lung cancer sample (Figure 2A). The trail lengths ranged from 6 to 9 spots, with an average span of 7 spots (~0.7 mm). Notably, some trails were observed to originate near blood vessels (Figure 2, B and C), while others predominantly traversed along loosened tissue regions or tumor necrosis regions (Figure 2D). We also observed similar patterns in trails identified in the ovarian cancer sample (Supplemental Figure 4, C–F; supplemental material available online with this article; https://doi.org/10.1172/jci.insight.192718DS1). These findings align with our hypothesis that T cells may preferentially navigate through less dense areas within the TME to avoid physical barriers such as abnormally dense collagen fibers. Of the 19 migration trails, 3 extended deep into the tumor core, with a median length of 0.7 mm. This suggests that T cells might rapidly become exhausted after relatively short journeys into the immunosuppressive TME.

### Elevated T cell chemotaxis along the migration trails.

**Figure 3:**
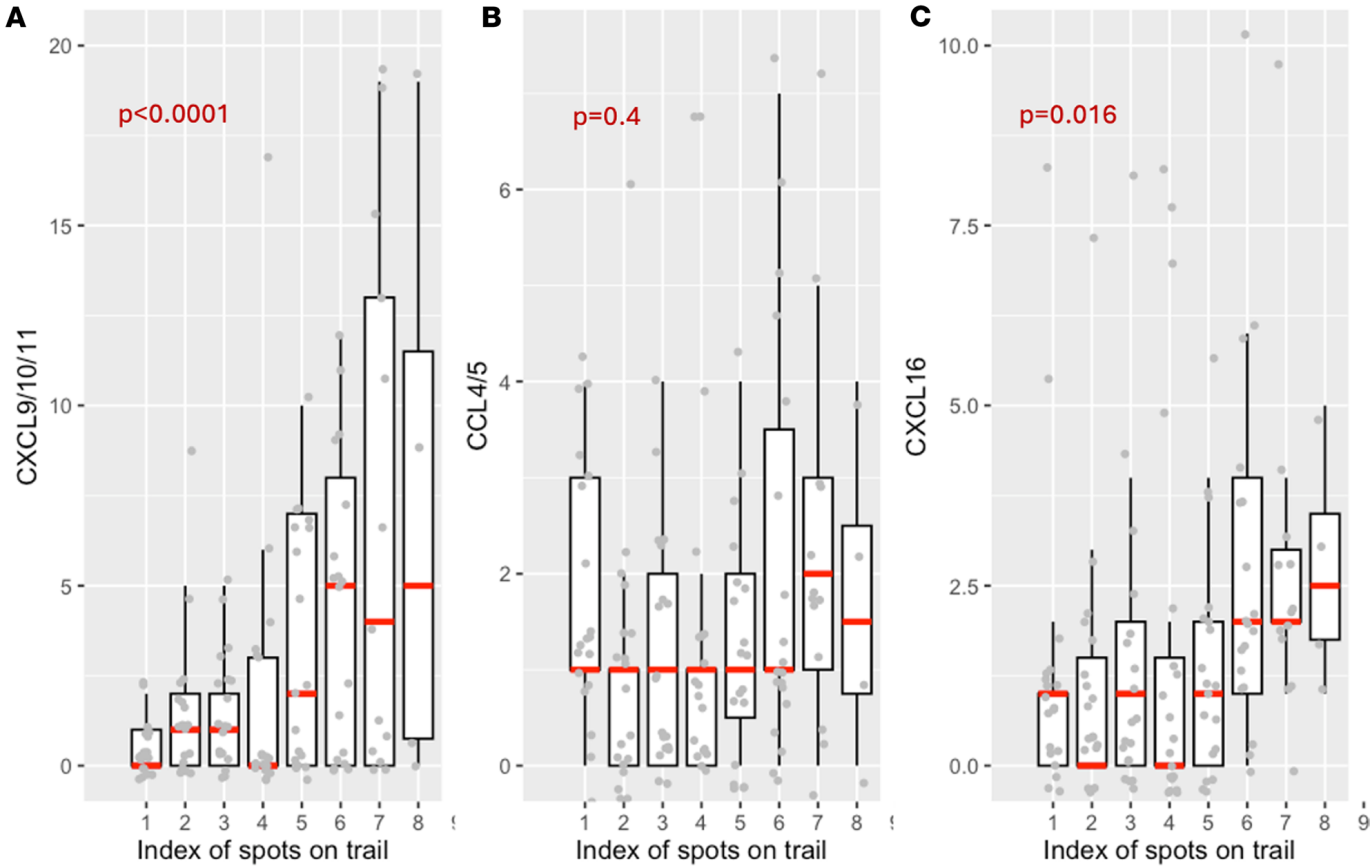
Trend of chemokines that promote T cell migration along the algorithm-identified migration trails, and phenotype of these trails identified in the lung cancer sample. (**A**)Total expression of CXCL9, CXCL10, and CXCL11 along the algorithm-identified migration trails, with the median of each index labeled with a red line segment. Mixed-effect linear regression adjusting for UMI of each spot was performed to test the increasing trend of ligand expression along the trails, and 2-sided *P* values are reported. (**B**) Total expression of CCL4 and CCL5 along the algorithm-identified migration trails, with the median of each index labeled with a red line segment. (**C**) Expression of CXCL16 along the algorithm-identified migration trails,with the median of each index labeled with a red line segment.

As experimental tracing of T cell migration in vivo is challenging, we sought to validate the trails through biological findings. Specifically, T cell migration is regulated by chemokine/receptors in the TME (6). Several key chemokine receptors are reported to direct T cell migration in the tumor (6, 28). Meanwhile, it is known that T cells respond to a positive gradient of the chemoattractants that guides the movement to the loci of interest (29, 30). We investigated the chemokines associated with putative T cell migration regulators, including *CXCL9/10/11/16* and *CCL4/5* along the migrating trails. These ligands have been reported to promote T cell infiltration into tumors (28). We observed a significantly increasing trend in expression for all the CXC motif chemokines for all the CXC motif chemokines, including *CXCL9/10/11* (ligand for *CXCR3*) and *CXCL16* (ligand for *CXCR6*), whereas the trend for *CCL4/5* was not significant in this lung cancer sample (Figure 3). *CXCR3* is one of the most well-studied receptors for T cell migration (31, 32) and has been implicated in antigen-specific T cell responses (33). No trend was observed for the chemokine receptors on the trails, possibly because the expression of these receptors is sufficient to induce T cell migration (6). We made similar observations in the melanoma sample and the ovarian tumor sample (Supplemental Figure 1).

### Shared TCR genes between spots on trail.

Next, we sought to provide another validation using a statistical approach. T cell antigen priming and proliferation occur in the draining lymph nodes (34) prior to the migration to the tumor site. Therefore, it is expected that T cells of the same clone are more likely to share a migration pathway. Here, as paired TCR hypervariable regions were not sequenced, we used the TCR α variable (*TRAV*) and joining (*TRAJ*) genes and the TCR β variable (*TRBV*) genes as surrogates to trace clonal sharing. We used an ovarian cancer sample for this analysis, as these genes were not sequenced in the other samples. To test whether spots on migration trails shared more TCR variable genes, we first obtained 1 matching trail for each of the algorithm-identified migration trails and called it 1 control set. We then obtained 5,000 such matched control sets (see Methods). An example of 1 control set is displayed in Supplemental Figure 2. The systematic analysis in this study was then based on comparing algorithm-identified migration trails with these matched control trail sets, rather than simply comparing spots on and off the migration trails. This approach accounts for the spatial distribution of gene expression, as requiring a “trail” to only consist of adjacent spots influences the expected gene expression pattern.

We calculated the mean number of shared TCR V/J genes among consecutive pairs of spots along each of the algorithm-identified trails. We then calculated the same statistics for each of the 5,000 control sets and got the empirical distribution of the median of the mean shared TCR V/J genes of each trail for each control set (Supplemental Figure 3A). This works as a surrogate for the shared TCR of each trail. The median number of the shared TCR V/J genes among the migration trails was significantly higher (median = 13.6) compared with the median in the empirical distribution of these statistics in control sets (median = 11.2), with the 2-sided empirical *P* value being 0.005. As different immune cells are reported to migrate together (6), we also investigated the clonal sharing of B cells, under the premise that the physical passages for T cell migration might also be utilized by B cells. Consistently, we observed that the median number of shared BCR variable genes among the 19 migration trails was significantly higher (median = 2.4) compared with the median in the empirical distribution (median = 1.9), with the 2-sided empirical *P* value being 0 (Supplemental Figure 3B). While these results may not provide direct validation, they supported our conclusion that the predicted trails were statistically enriched for true immune cell migration paths.

### Gene expression signatures along the migration trails.

**Figure 4:**
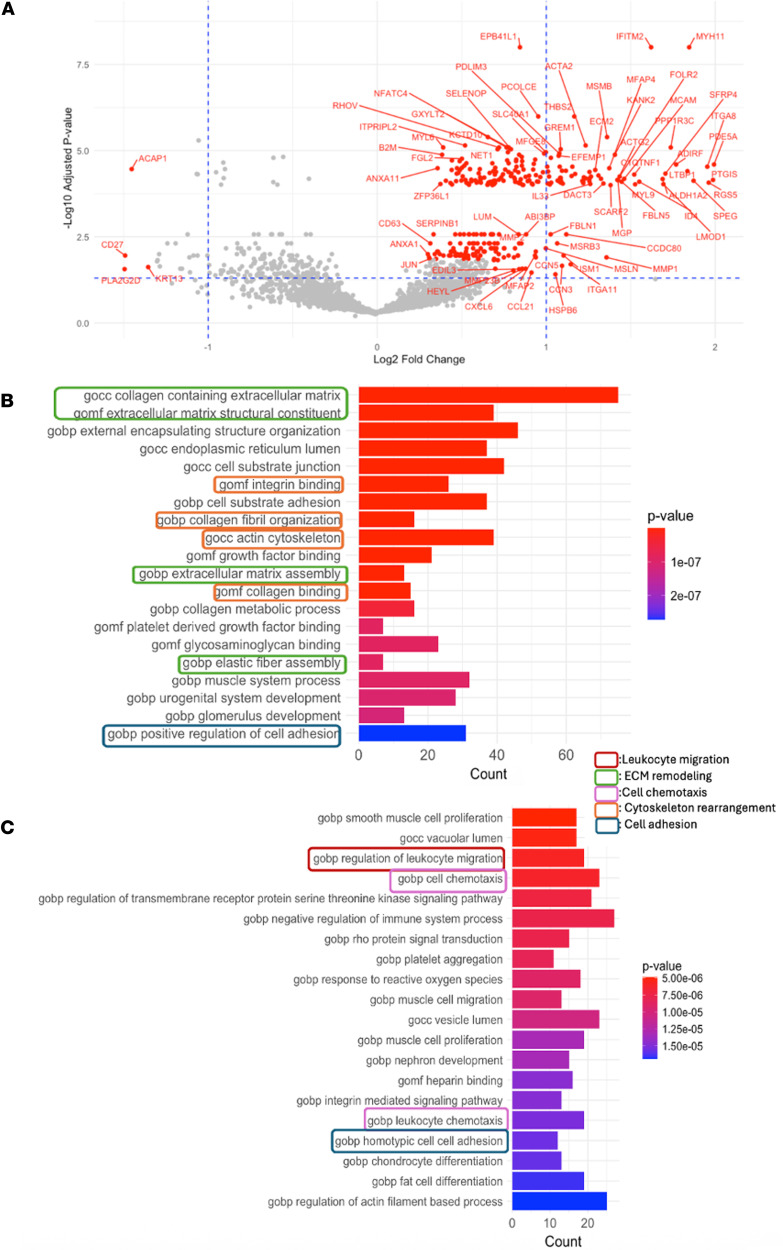
Differential gene expression analysis for T cell migration trails on the lung cancer sample. (**A**) Volcano plot of gene (3,000 variable genes) fold change (FC) and adjusted empirical *P* values between those on migration trails vs. 5,000 control sets. Genes with FDR <0.05 and the absolute values of FC ≥1.2 were labeled on the plot. Genes with –log_10_ *P* value >4 shown in this plot correspond to empirical *P* values of 0. For visualization purposes, these *P* values were replaced with randomly assigned values between 10^–4^ and 10^–8^ to enable plotting on a –log_10_ scale. (**B**) Bar plot showing the top 20 enriched Gene Ontology biological process (GOBP) gene pathways computed from the 394 significantly upregulated genes on migration trails by GSEA. Colors of the text box borders indicated the type of biological processes that may involved in T cell migration. Colors of the bars indicate the adjusted *P* values. Statistical significance was evaluated using Fisher’s exact test, with FDR corrected by the Benjamini-Hochberg approach. (**C**) Bar plot showing the top 50–70 enriched GOBP gene pathways computed from the 394 significantly upregulated genes on migration trails by GSEA.

We next investigated the gene expression signatures of cells on the migrating trails. We performed differential gene expression analysis by comparing the mean expression levels of the 3,000 variable genes in this lung cancer sample on the migration trails to the empirical distribution of gene expression generated with the 5,000 matched control trail sets. We identified 394 significantly upregulated genes (empirical FDR <0.05 and fold change >1.2, Figure 4A and Supplemental Table 1). Consistently, extracellular enzymes that break down the collagen network, including *MMP1* and *MMP2* (35), were significantly upregulated on the trails. In addition, genes encoding cell adhesion molecules that have been shown to promote cell migration and matrix remodeling were also upregulated on the migration trails, such as *ITGA8/11*, *TJP3*, etc. (36–39). Furthermore, we observed that *LGALS3*, which encodes Galectin-3, was significantly upregulated (adjusted *P* = 0.013). It is a ligand of *LAG3* that induces reduced T cell cytotoxicity and exhaustion (40). This gene independently emerged as a top hit in our analysis, where the *LAG3* level has been controlled. This result indicated that *LAG3*/Galectin-3 axis might be responsible for inducing T cell exhaustion along the trails. To understand the gene pathways related to T cell migration, we performed gene set enrichment analysis (GSEA) (41). As expected, the top hits contained many key components of cell migration (Figure 4, B and C). These pathways included the ones involved in the regulation of immune cell chemotaxis, cytoskeleton rearrangement, cell adhesion, and ECM remodeling.

These observations were validated by the ovarian tumor sample. Implementing the same trail-identification method, we found 29 migration trails, and most of them were in the tumor region (Supplemental Figure 4). The length of these trails varied between 6 and 8 spots, with the average being 6.6 spots, similar to the first sample. For the ovarian tumor sample, we also obtained 5,000 control trail sets. We analyzed the upregulated genes on the migration trails and identified a total of 191 genes that were overexpressed along these trails compared with the control trails (Supplemental Figure 5A and Supplemental Table 2). Importantly, we reproduced the observation that Galectin-3 was upregulated on the migration trails (adjusted *P* = 0.010). We also observed similar GSEA results with pathways enriched for immune cell migration (Supplemental Figure 5, B and C). We performed the same analysis for the melanoma sample and identified upregulated genes overexpressed along the migration trails (Supplemental Table 3).

### Reproducible observations of T cell migration trails on Visium HD samples.

**Figure 5:**
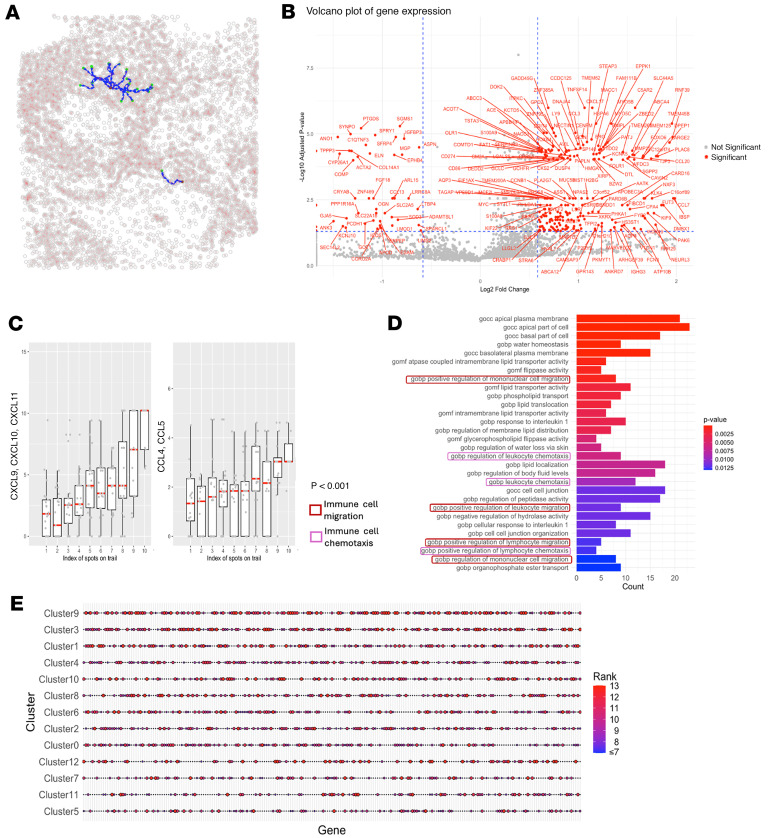
Algorithm-identified migration trails in the first HD lung cancer sample. (**A**) Algorithm-identified migration trails labeled as splines on the spatial coordinates, with the starting location marked as green. Gray spots represent spots with T cell infiltration, and red centers indicate spots with high levels of expression of lung cancer markers. (**B**) Volcano plot of gene (3,000 variable genes) fold change (FC) and adjusted empirical *P* values between those on migration trails vs. 5,000 control sets. Genes with FDR <0.05 and the absolute values of FC ≥1.5 were labeled on the plot. (**C**) Left: Total expression of CXCL9, CXCL10, and CXCL11 along the algorithm-identified migration trails, with the median of each index labeled with a red line segment. Mixed-effect linear regression adjusted for UMI of each spot was performed to test the increasing trend of ligand expression along the trails, and 2-sided *P* value is reported. Right: Total expression of CCL4 and CCL5 along the algorithm-identified migration trails, with the median of each index labeled with a red line segment. (**D**) Bar plot showing the top 35 enriched Gene Ontology biological process (GOBP) gene pathways computed from the 683 significantly upregulated genes on migration trails by GSEA. Colors of the text box borders indicated the type of biological processes that may involved in T cell migration. Colors of the bars indicate the adjusted *P* values. Statistical significance was evaluated using Fisher’s exact test, with FDR corrected by the Benjamini-Hochberg approach. (**E**) Bubble plot showing the enrichment of overexpressed genes on T cell migration trails in each of the T cell clusters in **A**.

To assess the reproducibility of ReMiTT findings in high-resolution ST data, we next applied ReMiTT to 2 lung tumor samples and 1 breast cancer sample generated using the Visium HD platform. We identified 21 trails in the first lung sample (HD-lung1; mean length 0.73 mm, Figure 5A), 9 trails in the second lung sample (HD-lung2; mean length 0.60 mm, Supplemental Figure 6A), and 62 trails in the breast cancer sample (HD-breast; mean length 0.74 mm, Supplemental Figure 7A). These characteristics of algorithm-defined T cell migration trails in HD datasets were highly concordant with those observed in standard Visium data. However, unlike migration trails in the low-resolution data, migration trails in the Visium HD datasets were concentrated within 1 or 2 localized regions of each tumor. This pattern was largely attributable to the higher dropout rate in HD data. Because trail detection requires nodes with detectable T cell signatures to be spatially adjacent to each other, regions with sparse T cell pixel presence cannot support trail formation under the current algorithm — and these sparsely infiltrated regions (due to high dropout) account for the majority of the HD tissue area. Nevertheless, in regions with denser T cell representation, the increased resolution of HD data allowed us to identify multiple distinct trails. These trails converged toward the same destination area, suggesting a potential entry point or focal zone of T cell infiltration into the TME.

We next investigated putative regulators for T cell migration. First, genes enriched along HD migration trails included chemotactic factors that can promote leukocyte recruitment, such as *CXCL11*, which was significantly elevated in both HD-lung1 and HD-breast samples (Figure 5B, Supplemental Figure 7B, and Supplemental Tables 4 and 6). We then comprehensively investigated the chemokine ligands involved in T cell guidance. Owing to the high dropout of *CXCL16*, we focused on *CXCL9/10/11* and *CCL4/5*. In the HD-lung1 sample, both the *CXCL9/10/11* axis and *CCL4/5* exhibited significant increasing trends along the trails (*P* < 0.001 for both; Figure 5C). Similar patterns were observed in HD-lung2 and HD-breast samples (Supplemental Figure 6C and Supplemental Figure 7C).

We also confirmed the elevated expression of genes involved in cell adhesion and ECM remodeling, including *ITGAL*, *ITGA3/4/5*, and *TJP3* (Figure 5B, Supplemental Figure 6B, Supplemental Figure 7B, and Supplemental Tables 4–6). At the gene set level, pathways related to leukocyte adhesion, lymphocyte migration, and leukocyte chemotaxis were among the top enriched categories across all HD samples, consistent with our findings using the Visium ST datasets (Figure 5D, Supplemental Figure 6D, and Supplemental Figure 7D). Together, these results further support the reliability of ReMiTT on identifying biologically meaningful T cell migration trails across multiple tumor types and ST platforms.

### Phenotype of on-trail migrating T cells.

**Figure 6:**
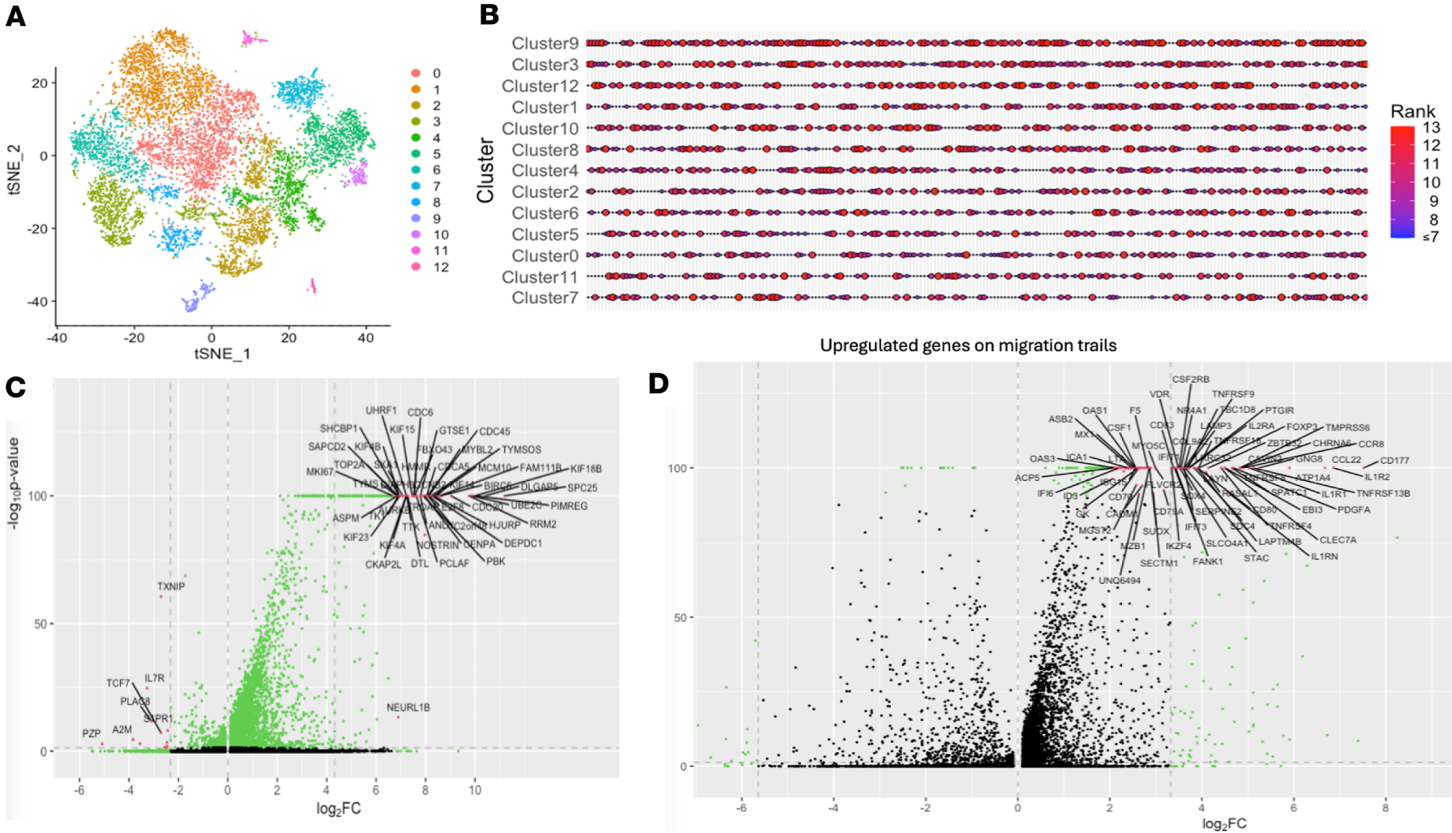
Phenotypic investigation of T cells on the migration trails. (**A**) t-SNE plot for the 11,236 T cells from deep single-cell RNA-seq data of 14 patients with non–small cell lung cancer, with the nearest-neighboring clusters color-labeled. (**B**) Bubble plot showing the enrichment of overexpressed genes on T cell migration trails in each of the T cell clusters in **A**. The large bubble sizes and red colors indicate higher ranks of mean expression of each of the upregulated genes on migration trails among all the 13 clusters of T cells. Clusters (*y* axis) were ordered by their median ranks of the genes. (**C**) Volcano plots of gene fold change and adjusted *P* values for cluster 9 compared with other clusters. Statistical significance was evaluated using the Wilcoxon’s rank-sum test with FDR corrected using the Benjamini-Hochberg procedure. (**D**) Volcano plots of gene fold change and adjusted *P* values for cluster 3 compared with other clusters. Statistical significance was evaluated using the Wilcoxon’s rank-sum test with FDR corrected using the Benjamini-Hochberg procedure.

We next sought to explore the phenotypes of the T cells on the migrating trails using single-cell RNA-seq data profiled from human T cells. Specifically, we obtained the data from a previous study of 12,346 T cells from 14 patients with non–small cell lung cancer (42). We used this dataset due to its high data quality and well-accepted T cell subset annotations. We performed unbiased clustering based on the top 30 principal components on these T cells and identified 13 clusters (Figure 6A). We compared genes upregulated on T cell migration trails identified in our lung cancer sample to investigate which cluster showed similar gene expression signatures. We observed that cluster 9 and cluster 3 were top enriched (Figure 6B and Supplemental Table 7). This analysis was repeated in other ST tumor samples (Supplemental Figure 8 and Supplemental Tables 8 and 9) and all the HD tumor samples (Figure 5E, Supplemental Figure 6E, Supplemental Figure 7E, and Supplemental Tables 10–12, Supplemental Figure 6E, and Supplemental Figure 7E); our results suggest that clusters 9 and 3 present the phenotype of the moving T cells on the migration trails.

T cells of cluster 9 expressed a high level of *MKI67* and several members of the kinesin family (Figure 6C). These T cells are likely proliferating T cells with massive cytoskeleton remodeling and intracellular protein trafficking. We also identified cytotoxic markers in the top-upregulated genes in this cluster, suggesting that it might contain cytotoxic CD8 T cells. Cluster 3 expressed high levels of Treg markers *FOXP3* and *IL2RA* (Figure 6D). These observations might be associated with a previously reported migrating Treg population (43).

This result also indicated that on the migration trails there may be a mixture of immune cell populations. Therefore, we also investigated TAMs, which are also known regulators of T cell antitumor responses (44). Consistently, we observed an increasing trend of macrophage markers along the migration trails in melanoma and lung cancer (Supplemental Figure 9). Together, our results might suggest a comigration of multiple immune cell types with T cells in the TME.

## Discussion

Tumor-infiltrating lymphocytes are the functional carriers of antitumor immunity. As T cells need to make physical contact with their target cells to perform the cytotoxic function, their successful infiltration into the tumor core is the basis of an effective immunotherapy of adoptive T cells (45–49). However, how T cells migrate within TME and how to improve their chance of reaching their targets are still understudied. Although a few in vitro studies provide valuable insights on how T cells migrate in 3D matrix structure, it is very difficult to track T cells within human tumor tissues (15, 16). Compared with normal tissues or any 3D matrix structures created in an in vitro experiment, ECM throughout tumors is highly dysregulated with substantial chemical and structural differences. Therefore, in vitro experiments on T cells migration may not accurately reflect this crucial part of antitumor immune response in human TME.

Using ST data, we developed what we believe to be a novel method to identify migration trails of T cells in vivo, under the assumption that T cells in the TME gradually increase their exhaustion levels. ReMiTT could offer an unbiased approach to investigate T cell migration in the tumor. When applied to multiple human cancer samples from both the 10x Visium and Visium HD ST platforms, we got consistent results between samples from the two platforms. In addition, we made the following observations: (a) several chemokines known to direct T cell migration exhibited an increasing trend along the trails; (b) the Lag3/Galection-3 axis is a potential regulator for T cell exhaustion during migration; and (c) the phenotype of the migrating T cells resembles that of previously reported T cell clusters with a mixture of proliferative markers and Treg signatures.

TAMs may play a positive or a negative role in antitumor immunity (50). For example, M1 macrophages are proinflammatory and tumor resistant, while M2 macrophages are antiinflammatory and protumor. While it is not feasible to distinguish the 2 cell types without single-cell resolution, we observed several signature genes of activated antitumor macrophages, including *FOLR2* (51), *PIM1*, and *SCL11A1* (52), which were upregulated on the migration trails. These observations might suggest a positive role of macrophages in T cell migration in the TME. However, it should be noted that further evidence will be needed to prove the physical interactions between TAMs and the migrating T cells.

Why is it possible to observe 3D migration trails on a thin-layer 2D slide? Most of the trails were found close to vessels or loose tissues, which are expected to be spherical, instead of flat. Therefore, it is possible that the trails are derived from the intersection between a 2D manifold and the cutting surface of the slide. The 2D manifold might be the thin gaps between blocks of tissues in the 3D space. If this is true, the picture of real T cell migration in the tumor might be much more complicated than previously thought, where T cells can choose different directions to move through. Generally speaking, finding 3D trails on the 2D plane requires the trails to be “nearly flat,” which can be relatively rare given the complex structure of the TME. The consequence of this limitation is reduced sensitivity, which explains why ReMiTT only reported a few trails, given the large amount of T cell–containing spots in each sample.

There are several limitations to this work. First, the Visium spatial RNA-seq data are not at the single-cell level. Each spot has a diameter of 55 microns, so it can cover 0–10 cells, depending on the spot’s location. Therefore, our analysis is based on the assumption that if multiple T cells are present in 1 spot, their exhaustion levels are similar. Second, the sensitivity of this method hinges on the high level of T cell infiltration and large span of T cell exhaustion markers, which may not be observed for all the tumor ST samples. Finally, the scope of this work remains exploratory, and most conclusions in this work remain descriptive. Future experiments with in vivo tracing of T cell trajectories will be needed to test the validity of migration trails identified by ReMiTT. Despite these limitations, findings in this study might provide insights into T cell migration and exhaustion in the TME and facilitate the improvement of cancer immunotherapies.

## Methods

### Sex as a biological variable

Sex was not considered as a biological variable in this study.

### T cell–infiltrated spots and exhaustion score

We only include spots with T cells for analysis in this study. We identify T cell–infiltrated spots using the expression of T cell surface markers *CD8A*, *CD8B*, *CD4*, *CD3D*, *CD3E*, and *CD3G*. We assigned a T cell exhaustion score for each of the T cell spots based on the expression of T cell exhaustion markers (*PDCD1*, *LAG3*, *HAVCR2*, *TIGIT*, *CTLA4*, *ENTPD1*, and *TOX*) (10, 53–60). To get exhaustion level of each spot while controlling for the T cell number in it, we performed linear regression with the dependent variable being the sum of gene expression of all these exhaustion markers in a spot and the independent variable being the sum of gene expression of all the T cell markers in the same spot, based on the assumption that spots with more T cells would have higher expression of T cell surface markers. Then, the T cell exhaustion score of each T cell spot was defined as the residuals of this linear model, reflecting how exhausted the T cells are in this spot after controlling for the number of T cells in it. For convenience, we shifted all the residuals to the right until the smallest exhaustion score was 0.

### Assignment of pixels to subclusters (HD data)

To mitigate high dropout and preserve spatial continuity in Visium HD datasets, we grouped adjacent spots with similar exhaustion scores into local subclusters, referred to as nodes. We applied a region-growing greedy clustering algorithm as follows. (a) We selected an unassigned pixel as a seed and (b) identified all neighboring pixels within a fixed spatial radius (e.g., 5× pixel size). (c) Among these neighbors, we assigned to the same node those with exhaustion scores that fell within a very small similarity tolerance, (d) marked all assigned spots, and removed them from future consideration. (e) Steps 1–4 were repeated until all spots had been assigned to a node.

This procedure yielded a set of spatially coherent nodes that represent local neighborhoods of similar exhaustion states, enabling ReMiTT to operate on biologically meaningful units despite sparsity in the HD data.

### Potential start and end nodes of trails

Assuming that activated T cells experience gradual exhaustion as they navigate through the TME, the starting point of a migration trail would be a T cell node with a low exhaustion score, while the endpoint would be a node with a high exhaustion score. We ranked all T cell nodes based on their exhaustion scores and selected the first 33% of nodes with the lowest exhaustion scores as the potential start points and the last 33% with the highest exhaustion scores as the potential endpoints. With these potential start and endpoints, we identified potential migration trails with two methods.

### Minimum-spanning path

The first method of trail identification attempted to connect start points to endpoints using Dijkstra’s algorithm (61). In this method, we defined a directed, weighted graph G(V,E) with all the T cell nodes and their spatial locations. In this graph, *V* stands for vertices, in our case, all the T cell nodes. *E* stands for edges. The 10x Visium spatial gene expression data were organized in a hexagonal grid. Each spot (except those at the boundary of the slide) had 6 neighboring spots, with the distance between adjacent spots being approximately 110 microns. On graph G, edges only existed between neighboring nodes, with 2D distances smaller than 130 microns. In other words, we did not allow for spatial gaps along consecutive pairs along the migration trail. The direction of edges was set to point from the node with the smaller exhaustion score to the node with the larger exhaustion score. Then, if a pair of potential start and end nodes could be connected on graph G by a few intermediate spots, we were able to find a potential migration trail, with T cells gradually getting more and more exhausted. It is possible that 2 nodes can be connected by multiple routes. Among these routes, we chose the 1 with gradual changes in T cell exhaustion stage rather than having big jumps in exhaustion stages between consecutive pairs. Thus, we assigned the weight of edges to be the difference in exhaustion score between the 2 spots and used Dijkstra’s algorithm to find the shortest spanning path based on the weights of edges (62, 63). For each of the pairs of potential start and end nodes, we applied this method and got potential T cell migration trails. Then, we excluded trails featuring short distances between start and end nodes (<500 microns) and trails that comprised fewer than 5 nodes.

### Line in 3D space

The minimum spanning path method, while helping to identify trails on which T cells have gradually changing exhaustion stages, requires exhaustion score to be strictly increasing along the trail. The exhaustion score in this study was a rough measure on T cell exhaustion stage. To require it to be strictly increasing along the way might exclude some of the real trails. Thus, we developed the 3D line method to release this limitation and allow small fluctuations on exhaustion scores along the route while making sure the trend is still an increasing one.

For each T cell node, we use *x* and *y* to represent its 2D coordinates on the tissue slide and *z* to represent its exhaustion score. Consequently, the 3D coordinates formed by *x*, *y*, and *z* of the T cell nodes along a migration trail would approximate a segment of a straight line in this 3D space for those T cells migrating roughly in a linear pattern. To get this line segment, we first rescaled the *xy* coordinates of T cell nodes based on the minimum and maximum value of the exhaustion score (*z* coordinate) in this data sample. After rescaling, the range of both *x* and *y* coordinates became the same as the range of *z* coordinates. Then, for each pair of potential start and end nodes, we constructed a straight line in the 3D space connecting these 2 nodes. We then calculated the 3D distance between all other T cell nodes and this 3D line and kept the ones that have a very small distance to this line as a potential migration trail. The cutoff point was set arbitrarily, but we advise users to use a small value. We then determined the indexing of nodes along the trail based on projections of each node to this straight line, beginning from the end with the smaller exhaustion scores.

Similar to the minimum spanning path method, we only included trails with at least 5 nodes as potential migration trails and excluded trails with start and end node too close to each other. Furthermore, we only kept trails with all the T cell nodes adjacent to each other (≤130 microns in 2D [*xy*] distance). Finally, trails with large drawbacks between any consecutive pairs of nodes in exhaustion score along the trail were excluded.

### Trail selection

Using both methods, we obtained potential T cell migration trails. We then finalized migration trails, assuming that as a stream of T cells migrated through loosened collagen fibers, their expression of T cell stage–related markers would have a higher correlation compared with T cells off the migration trails. To make this comparison, we first obtained 5–15 alternative routes between the same start and end point for each potential migration trail (Figure 1). For the alternative trails, there was no requirement for an increasing exhaustion score along the way, but all the other criteria for a potential migration trail applied, such as, consecutive pairs of nodes along the trail must be neighbors, a trail must have ≥6 spots, and all the spots on alternative trails are T cell–infiltrated spots.

Among markers indicating T cell stages (42), we selected the ones that were significantly positively correlated with the level of expression of T cell markers for this filtering. For each migration trail, we calculated the mean pairwise correlation of the normalized expression level of the selected T cell stage–related genes (64). We then calculated the mean pairwise correlation for each of its alternative routes. The final set of migration trails all met two criteria: (a) mean pairwise correlation larger than all of its own alternative routes and (b) mean pairwise correlation larger than 85th percentile of all alternative routes.

### Systematic analysis of algorithm-identified migration trails

#### Testing the trend of cytokine and chemokine receptors along the algorithm-identified migration trails.

We assigned an index to each spot along the migration trails identified by our algorithm with the starting spot’s index being 1. For *CXCL9/10/11*, we calculated the total expression of these ligands and performed a mixed linear regression analysis. In this model, the total expression of these ligands (normalized for UMI of each spot) served as the dependent variable, while the independent variables were the indices of spots on each trail. Migration trails were treated as random effects. A positive β coefficient for the spot indices, accompanied by a *P* value smaller than 0.05, indicated a significantly increasing trend in the expression of these chemokines along the migration trail. The same analysis was performed for *CCL4/5* and *CXCL16*. These analyses were performed with the lung cancer sample, ovarian cancer sample, and melanoma sample.

Similarly, we tested the trend of markers of macrophages (total expression of *CD68*, *CD163*, *CD80*, *CD14*) along the migration trails (65, 66) with this method.

#### Assigning matched control trails for each algorithm-identified migration trail.

We first performed clustering analysis with 3,000 variable genes in the spatial data and divided the spots into 6 clusters based on dimensionality reduction methods (67). Then, for a migration trail, 1 matched control trail was generated with the same length as the migration trail and within the same cluster where the migration trail belongs. Additional criteria for migration trail identification were also applied when generating the control trail, including requiring consecutive pairs along the trail to be adjacent to each other and all spots on the trail need to be T cell spots, and ensuring that the spatial distance between the start and end spots of a trail were ≥0.5 mm. For each of the *n* migration trails defined by the algorithm, we got 1 matched control trail. We call these *n* control trails 1 control set. We generated 5,000 matched control sets for each tumor sample.

#### Shared TCR clones along trails compared with control trails.

The test of whether T cells on migration trails were more likely to descend from the same clone compared with T cells not on these trails could only be performed in 1 ovarian tumor sample, as the other samples did not have TCR variable genes. For each of the 25 migration trails identified in this sample, we calculated the mean number of shared TCR variable genes between consecutive pairs on the trails.

Specifically, for each trail, we examined the number of shared TCR variable genes between neighboring pairs of spots along the trail. The mean number of shared TCR variable genes for each trail was then calculated by averaging these values. The median number of shared TCR variable genes of the 25 migration trails was then compared with the empirical distribution calculated by the 5,000 control sets, and we reported the 2-sided empirical *P* values. We performed the same analysis for BCR variable genes, as B cells might as well migrate along these “physical highways,” and if this is the case, they could be sharing more BCR variables compared with those on control trails.

#### GSEA.

We used transcripts per million for gene expression in each spot to systematically analyze observed trails. To identify upregulated genes and gene pathways on these identified migration trails, we calculated the mean expression for each of the 3,000 variable genes among migration trails and control sets. We then calculated empirical *P* values for each gene using the empirical distribution generated by the 5,000 control sets. Benjamini-Hochberg adjustment was performed on these *P* values to account for multiple comparisons. With genes significantly overexpressed on migration trails compared with the control sets, we performed GSEA (41). We used the C5 collection GO term of Human Molecular Signature Dataset (MSigDB) for this analysis (68). GO term contains 10,402 gene pathway sets that are related to oncology.

#### Phenotype of migrating T cells.

We then characterized the phenotype of the moving T cells on our migration trails using deep single-cell RNA-seq data from 12,346 T cells from 14 patients with non–small cell lung cancer (42). Initially, we performed clustering analysis and divided these T cells into 13 clusters using *Seurat* (67). Specifically, unsupervised clustering was performed by first constructing a shared nearest-neighbor graph based on cell-cell similarity in a reduced dimensional space (PCA), and then, community detection was applied to identify groups of transcriptionally similar cells. Next, we calculated the mean expression for each of the significantly upregulated genes on our migration trails within each cluster. For each gene, we ranked the 13 means from smallest to largest, with rank 13 corresponding to the largest mean among the clusters. The T cell clusters with more top ranks indicate a higher enrichment of markers from our migration trails, suggesting that these clusters represent the phenotype of the migrating T cells on the identified trails.

### Statistics

In this study, we used a linear mixed-effects regression model to assess the significance of increasing trends in the density of T cell migration–promoting chemokine genes. We additionally applied 2-sided (2-tailed) *t* tests to identify genes upregulated along migration trails compared with control trails. Statistical significance was defined as *P* < 0.05.

### Study approval

This study analyzed only publicly available, deidentified datasets from 10x Genomics and GEO (GSE99254); no new human or animal samples were collected. Institutional review board approval was therefore not required.

### Data availability

We analyzed 3 publicly available ST data sets. Raw count matrices, images, and spatial information for 2 ST data sets from 10x Visium are accessible on the 10x Genomics website (https://www.10xgenomics.com/datasets and https://www.10xgenomics.com/datasets?configure%5BhitsPerPage%5D=50&configure%5BmaxValuesPerFacet%5D=1000&query=HD; 10x Visium Spatial Gene Expression ST samples: human lung cancer, human ovarian cancer, and human melanoma; Visium HD Spatial Gene Expression: human lung cancer sample 1, Visium HD Spatial Gene Expression, human lung cancer sample 2, Visium HD Spatial Gene Expression, and human breast cancer). The deep sequencing single-cell data for T cells can be downloaded from GEO (https://www.ncbi.nlm.nih.gov/geo/query/acc.cgi?acc=GSE99254).

An open-source implementation of the algorithm in R is available at https://github.com/zhonglin1234/ReMiTT

## Author contributions

Conceptualization: LZ, BL, QL, and GX. Methodology: LZ, QL, ZC, and GX. Investigation: LZ, BL, QL, GX, ZC, and SZ. Visualization: LZ, QL, ZC, and GX. Supervision: QL and GX. Writing of the original draft: LZ, BL, QL, GX, and SZ. Review and editing of the manuscript: LZ, BL, QL, GX, ZC, and SZ.

## Conflict of interest

The authors have declared that no conflict of interest exists.

## Funding support

This work is the result of NIH funding, in whole or in part, and is subject to the NIH Public Access Policy. Through acceptance of this federal funding, the NIH has been given a right to make the work publicly available in PubMed Central.

- National Science Foundation (2210912, 2113674) (to QL).
- NIH (1R01GM141519) (to GX and QL).
- NIH (R01GM140012, U01CA249245) (to GX).
- Cancer Prevention and Research Institute of Texas (CPRIT RP230330) (to GX).
- National Cancer Institute R01 grants CA258524 (to BL) and CA245318 (to BL).

## Supplementary Material

Supplemental data

Supplemental tables 1-12

Supporting data values

## Version 1 05/08/2026

Electronic publication
