# Supplementary material for: Computational identification of migrating T cells in spatial transcriptomics data
Source: JCI Insight. 2026 May 8;11(9):e192718. doi: 10.1172/jci.insight.192718 (PMC13232020; doi:10.1172/jci.insight.192718)
Supplement: Supplemental data [file jciinsight-11-192718-s218.pdf]

Supplementary Figures

Fig S1. Trend of chemokines that promote T cell migration along the algorithm-identified migration trails in melanoma and ovarian cancer samples

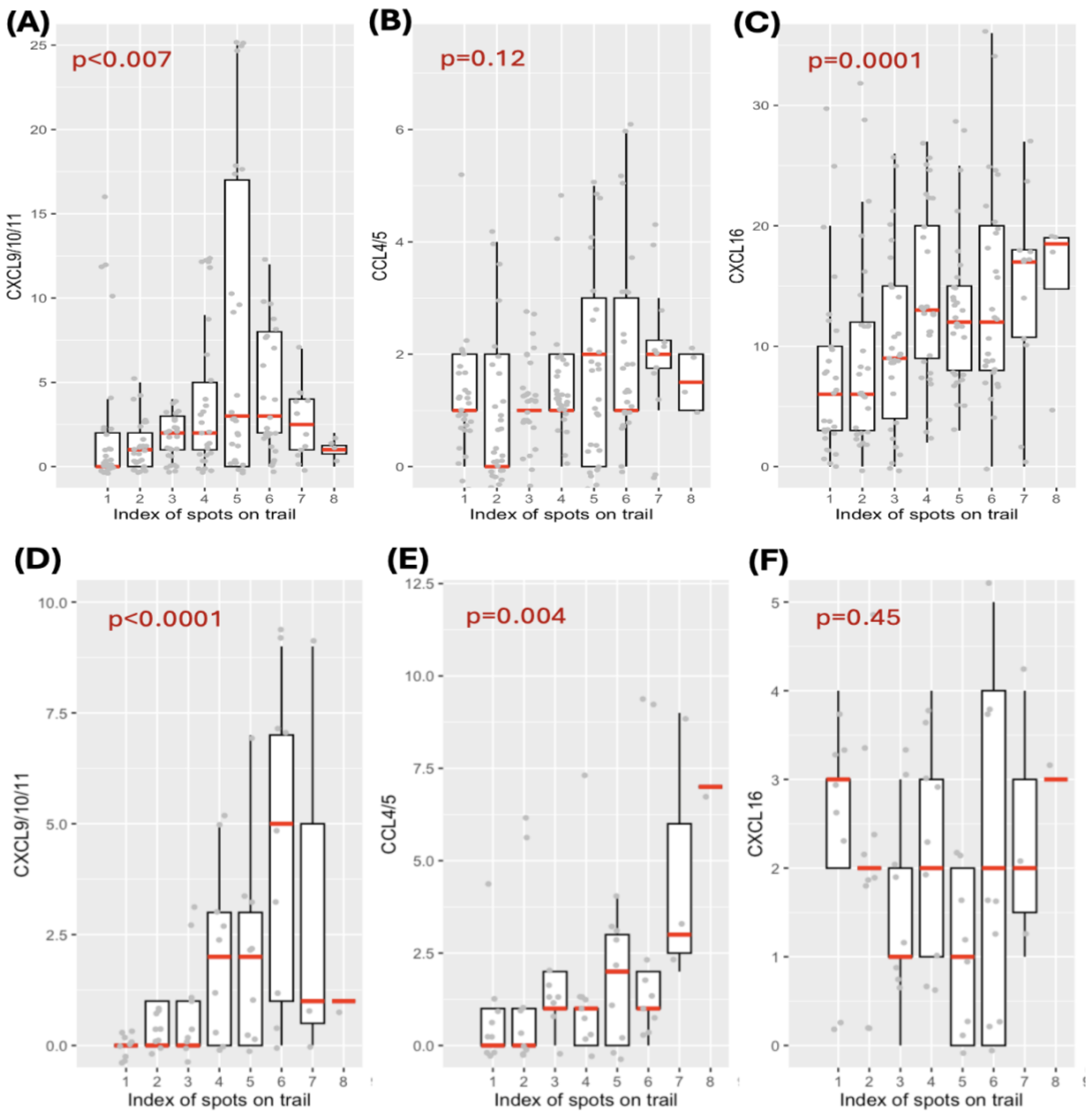

(A)-(C) Total expression of CXCL9, CXCL10, and CXCL11, Total expression and CCL4 and CCL5 and expression of CXCL16 along the algorithm identified migration trails in the **ovarian cancer sample** with the median of each index labeled with a red line segment. Mixed-effect linear regression adjusting for UMI of each spot is performed to test the increasing trend of ligand expression along the trails, and 2-sided p value is reported in the panel. **(D)-(F)** Total expression of CXCL9, CXCL10, and CXCL11, Total expression and CCL4 and CCL5 and expression of CXCL16 along the algorithm identified migration trails in the **melanoma sample** with the median of each index labeled with a red line segment. Mixed-effect linear regression adjusting for UMI of each spot is performed to test the increasing trend of ligand expression along the trails, and 2-sided p value is reported in the panel.

Fig. S2. An example of a matched control set paired with algorithm-identified migration trails

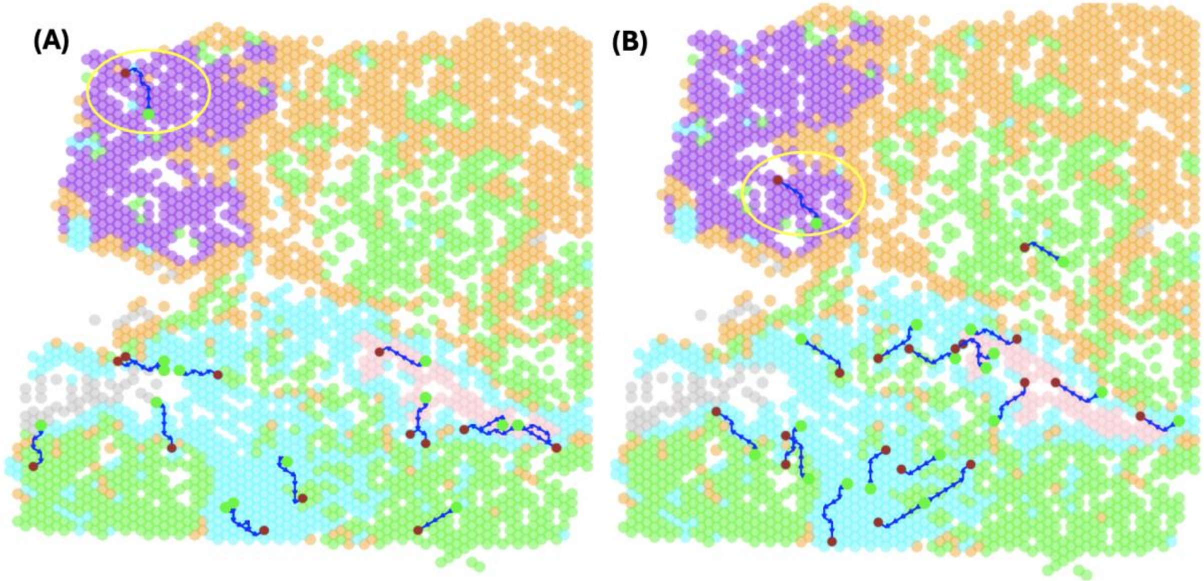

(A) The 19 algorithm-identified migration trails. **(B)** One matched control set-matched trails corresponding to each of the migration trails. Colored spots represent different gene-expression clusters. On each trail, the green spot marks the start point, and the brown spot indicates the end point. Each algorithm-identified trail is paired with a control trail of the same gene-expression cluster and length. All control trails are generated with T cell spots only, same as the migration trails. The circled pair of trails shows an example of an algorithm-identified trail and its corresponding control trail. While (B) shows one control trail set, a total of 5,000 control sets were generated for systematic analysis.

Fig. S3. Comparison of shared TCR and BCR variable genes between algorithm-identified trails and control trails in an ovarian tumor sample with TCR and BCR variable genes

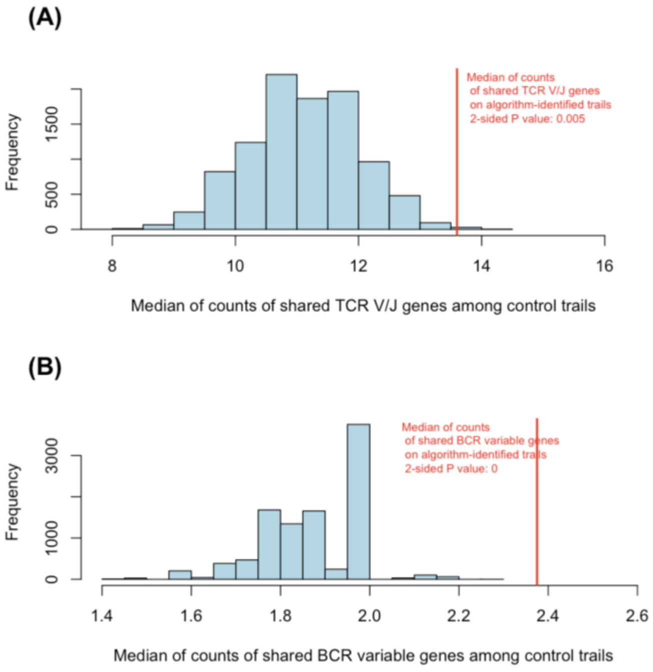

(A) The histogram shows the empirical distribution of the median of mean counts of shared TCR V/J genes between consecutive pairs within each control trail in a 25-trail set for 5,000 such control trail sets. The red line indicates the median of mean counts of shared TCR V/J genes for the 25 algorithm-defined migration trails (median = 13.6). The two-sided p-value for this comparison is 0.005. **(B)** The histogram shows the empirical distribution of the median of mean counts of shared BCR variable genes between consecutive pairs within each control trail in a 25-trail set for 5,000 such control trail sets. The red line marks the median count of shared BCR variable genes for the algorithm-defined migration trails (median = 2.375). The two-sided p-value for this comparison is <0.0001

Fig S4. Migration trails identified in a human ovarian cancer sample and their projections on the pathology slide.

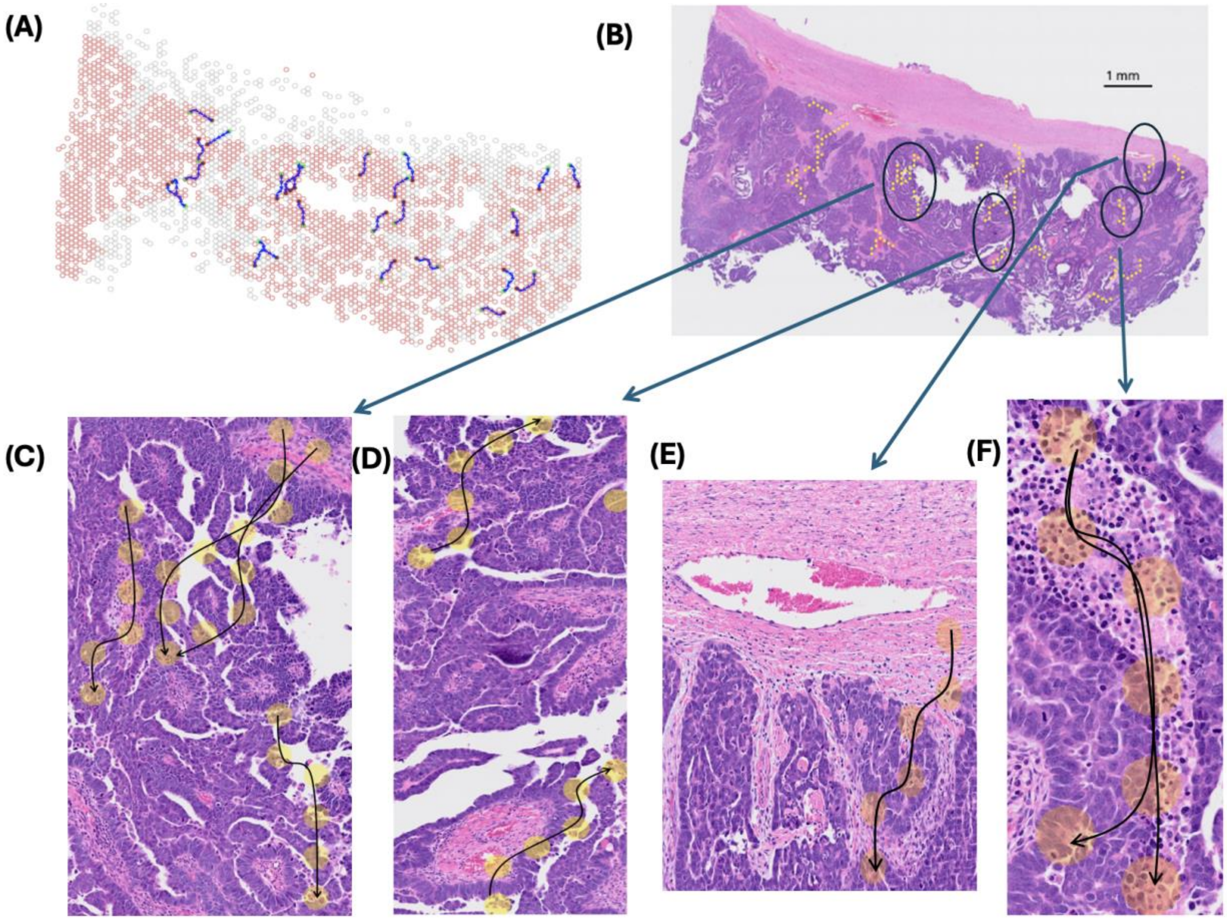

(A) Algorithm-identified migration trails labeled as splines on the spatial coordinates, with the starting location marked as green. Grey spots are spots with T cell infiltration and red circles indicating spots with high levels of expression of ovarian cancer markers. **(B)** Same trails overlaid on the pathology (H&E) slide of the tumor. **(C)** Examples of trails travel in less dense TME. **(D) and (E)** Examples of trails whose origin is close to vessels. **(F)** Moving T cells along tumor necrosis

Fig. S5. Differential gene expression analysis for T cell migration trails in the ovarian cancer sample

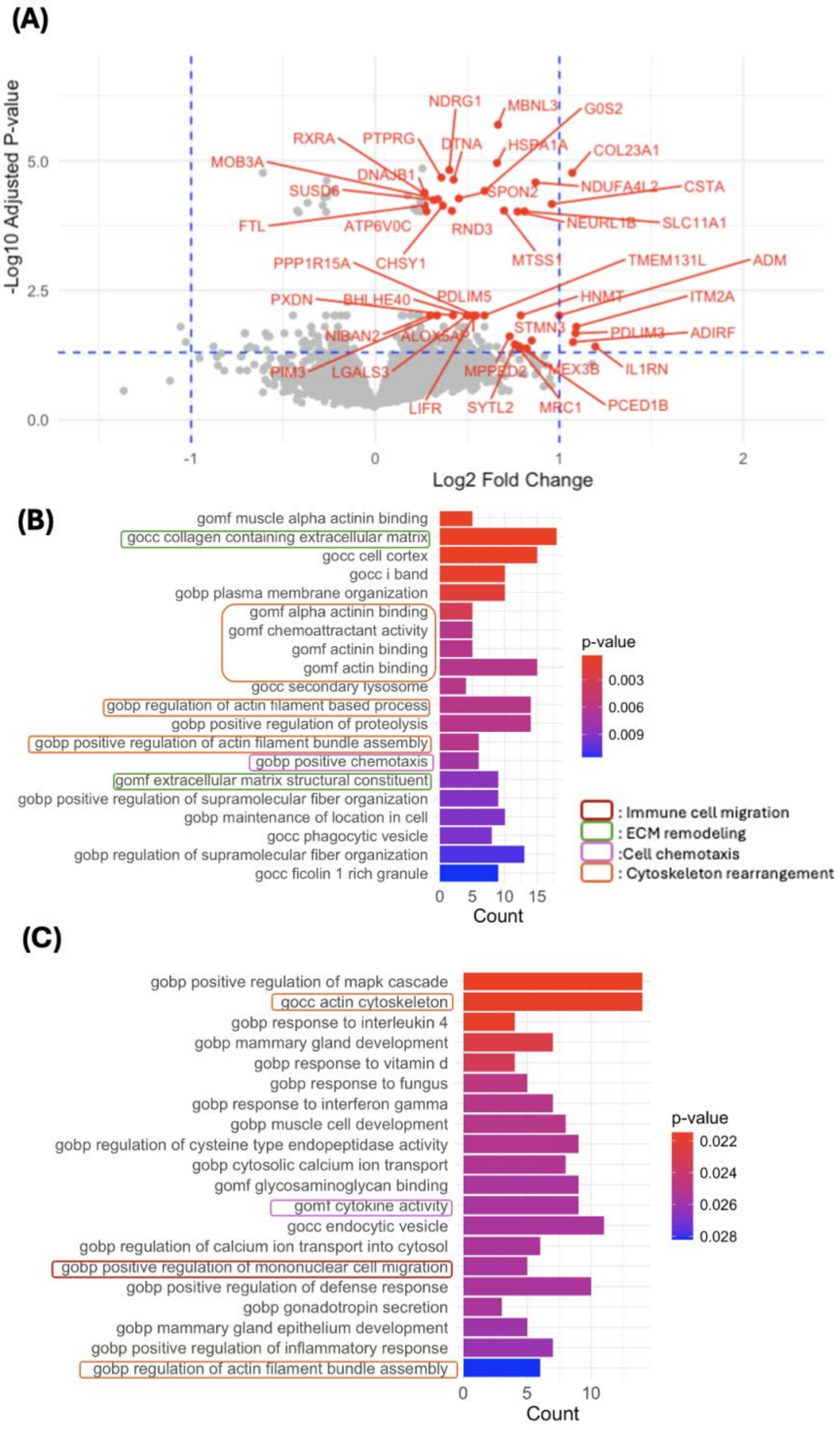

(A) Volcano plot of gene (3000 variable genes) fold change (FC) and adjusted empirical p values between those on migration trails vs. 5000 control sets. Genes with FDR<0.05 and the absolute values of FC ⩾1.2 were labeled on the plot. **(B)** Barplot showing the top 20 enriched Gene Ontology biological process (GOBP) gene pathways computed from the 191 significantly upregulated genes on migration trails by

GSEA. Colors of the textbox borders indicated the type of biological processes that may involved in T cell migration. Colors of the bars indicate the adjusted p values. Statistical significance was evaluated using Fisher's exact test, with FDR corrected by the Benjamini-Hochberg approach. **(C)** Barplot showing the top 40-60 enriched Gene Ontology biological process (GOBP) gene pathways computed from the 191 significantly upregulated genes on migration trails by GSEA.

Fig. S6. Algorithm-identified migration trails in the first HD lung cancer sample

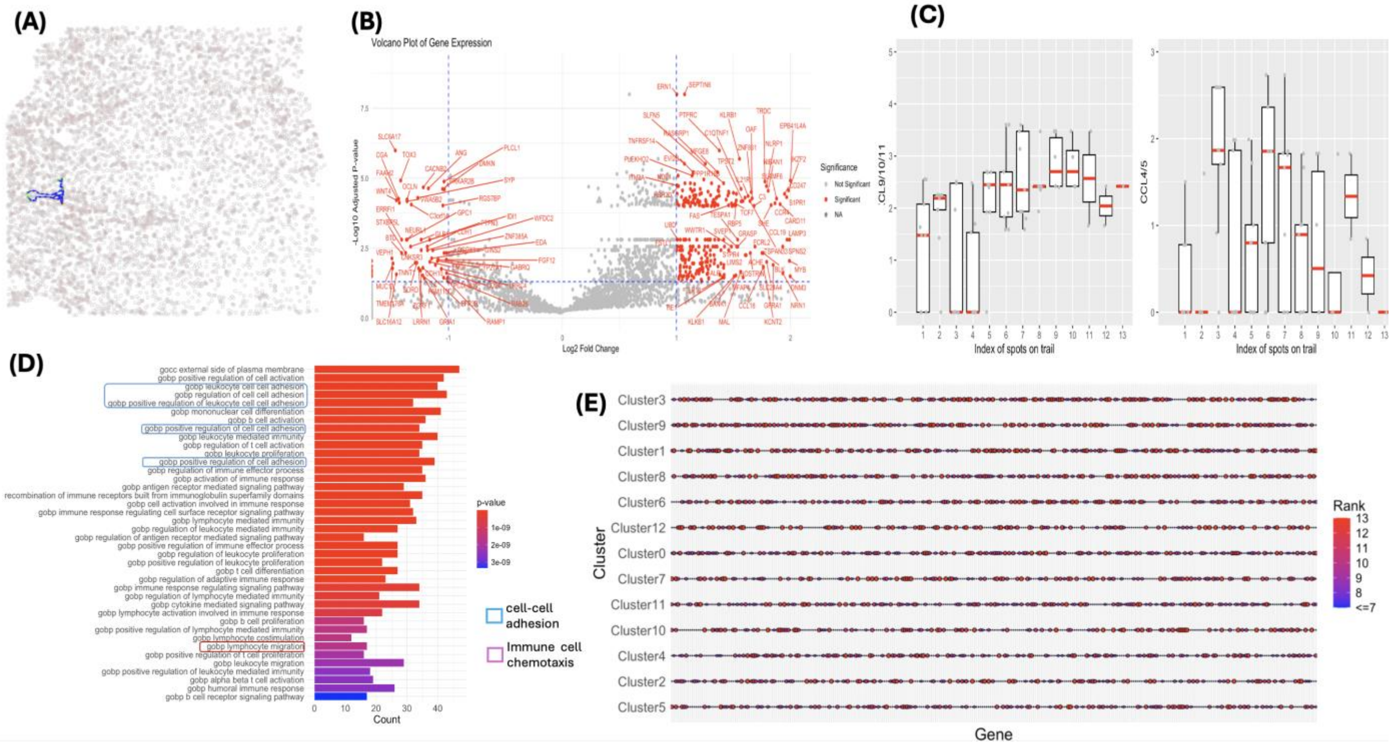

(A) Algorithm-identified migration trails labeled as splines on the spatial coordinates, with the starting location marked as green. Grey spots are spots with T cell infiltration, and red centers indicating spots with high levels of expression of lung cancer markers. **(B)** Volcano plot of gene (3000 variable genes) fold change (FC) and adjusted empirical p values between those on migration trails vs. 5000 control sets. Genes with FDR<0.05 and the absolute values of FC ⩾2 were labeled on the plot. **(C)** Barplot showing the top 35 enriched Gene Ontology biological process (GOBP) gen pathways computed from the 273 significantly upregulated genes on migration trails by GSEA. Colors of the textbox borders indicated the type of biological processes that may involved in T cell migration. Colors of the bars indicate the adjusted p values. Statistical significance was evaluated using Fisher's exact test, with FDR corrected by the Benjamini-Hochberg approach. (**D**) Left panel: total expression of CXCL9, CXCL10, and CXCL11 along the algorithm identified migration trails with the median of each index labeled with a red line segment. Mixed-effect linear regression adjusting for UMI of each spot is performed to test the increasing trend of ligand expression along the trails, and 2-sided p value is reported in the panel. Right panel: total expression of CCL4 and CCL5 along the algorithm identified migration trails, with the median of each index labeled with a red line segment. (**E**) Bubble plot showing the enrichment of overexpressed genes on T cell migration trails in each of the T cell clusters in Fig.5 (A).

Fig. S7. Algorithm-identified migration trails in the HD breast-cancer sample

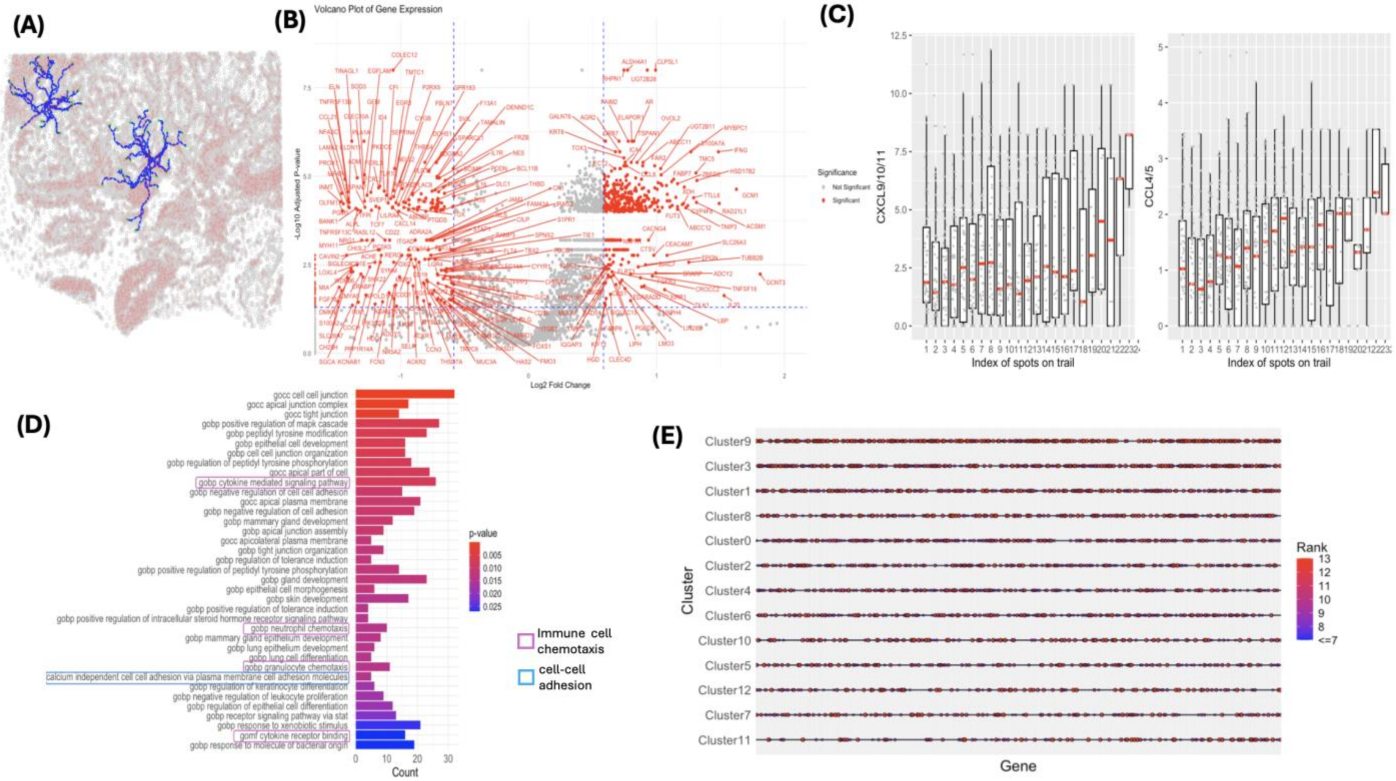

(A) Algorithm-identified migration trails labeled as splines on the spatial coordinates, with the starting location marked as green. Grey spots are spots with T cell infiltration, and red centers indicating spots with high levels of expression of lung cancer markers. **(B)** Volcano plot of gene (3000 variable genes) fold change (FC) and adjusted empirical p values between those on migration trails vs. 5000 control sets. Genes with FDR<0.05 and the absolute values of FC $\geqslant 1.5$ were labeled on the plot. **(C)** Barplot showing the top 35 enriched Gene Ontology biological process (GOBP) gen pathways computed from the 432 significantly upregulated genes on migration trails by GSEA. Colors of the textbox borders indicated the type of biological processes that may involved in T cell migration. Colors of the bars indicate the adjusted p values. Statistical significance was evaluated using Fisher's exact test, with FDR corrected by the Benjamini-Hochberg approach. (**D**) Left panel: total expression of CXCL9, CXCL10, and CXCL11 along the algorithm identified migration trails with the median of each index labeled with a red line segment. Mixed-effect linear regression adjusting for UMI of each spot is performed to test the increasing trend of ligand expression along the trails, and 2-sided p value is reported in the panel. Right panel: total expression of CCL4 and CCL5 along the algorithm identified migration trails, with the median of each index labeled with a red line segment. (**E**) Bubble plot showing the enrichment of overexpressed genes on T cell migration trails in each of the T cell clusters in Fig.5 (A).

Fig S8. Enrichment of overexpressed genes on T cell migration trails in each of the T cell clusters in the Lung single-cell cohort for ovarian cancer sample and melanoma sample

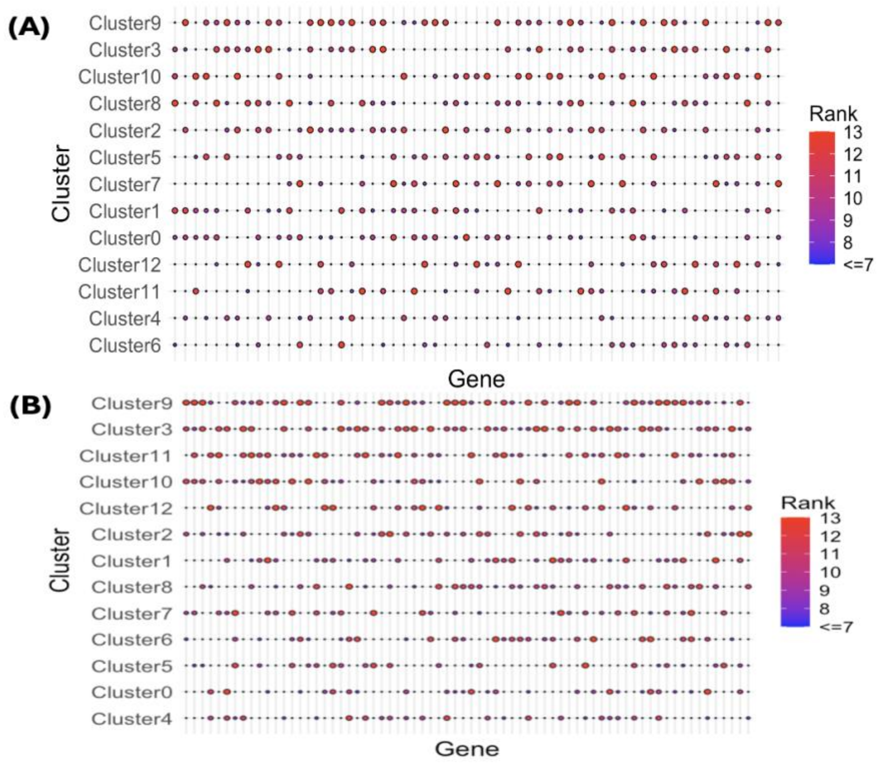

(A) Bubble plot showing the enrichment of overexpressed genes on T cell migration trails in each of the T cell clusters in Figure 5(A) in the ovarian cancer sample. The large bubble sizes and red colors indicate higher ranks of mean expression of each of the upregulated genes on migration trails among all the 13 clusters of T cells. Clusters (y-axis) were ordered by their median ranks of the genes. **(B)** Bubble plot showing the enrichment of overexpressed genes on T cell migration trails in each of the T cell clusters in Figure 5(A) for the melanoma sample.

Fig. S9. Trend of the expression of macrophage markers along the algorithm-identified migration trails

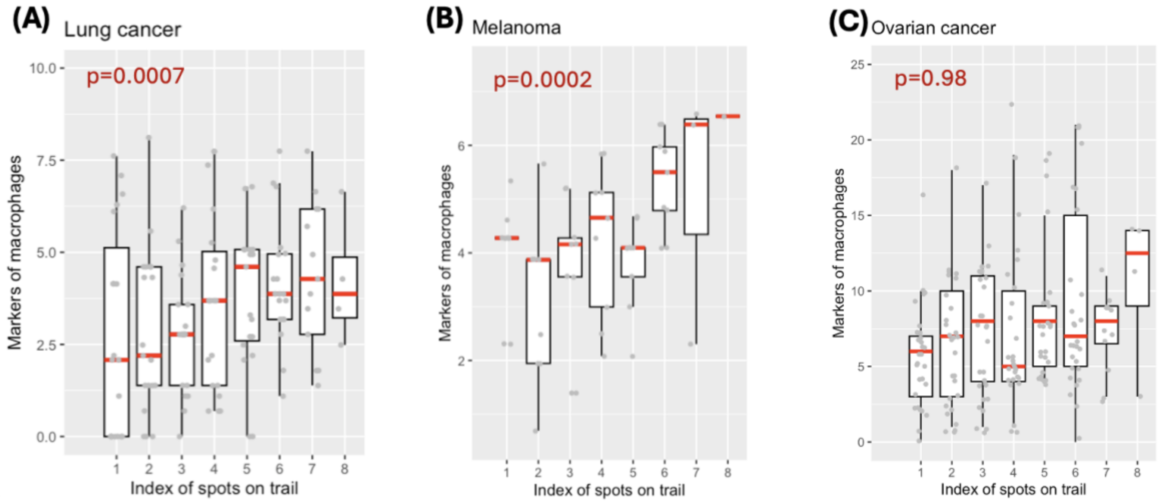

Total expression of macrophage markers (CD68, CD163, CD80, CD14) along the algorithm identified migration trails for the sample of lung cancer **(A),** melanoma **(B),** and ovarian cancer **(C),** with the median of each index labeled with a red line segment. Mixed-effect linear regression adjusting for UMI of each spot is performed to test the increasing trend of the expression along the trails, and 2-sided p values are reported in the panel.
